# High hepatitis E virus prevalence in pig slurry samples from the north-western region of Germany

**DOI:** 10.1016/j.onehlt.2025.101291

**Published:** 2025-12-08

**Authors:** Florian Hinte, Marc Lütgehetmann, Toni Luise Meister, Katja Giersch, Maura Dandri, Julian Schulze zur Wiesch, Sven Pischke

**Affiliations:** a Department of Internal Medicine, University Medical Center Hamburg-Eppendorf, Hamburg, Germany; b German Center for Infection Research (DZIF), Hamburg-Lübeck-Borstel-Riems, Germany; c Department of Medical Microbiology, Virology and Hygiene, University Medical Center Hamburg-Eppendorf, Hamburg, Germany; d Institute for Infection Research and Vaccine Development (IIRVD), Center! for Internal Medicine, University Medical Center! Hamburg-Eppendorf (UKE), Hamburg, Germany; e Department for Clinical Immunology of Infectious Diseases, Bernhard Nocht Institute for Tropical Medicine, Hamburg (BNITM), Germany

**Keywords:** HEV, Pig slurry, Density gradient, Zoonotic

## Abstract

Pork is recognized as the primary source of transmission for Hepatitis E virus (HEV) genotypes 3 and 4 (gt3/4) in industrialized countries. In Germany, approximately 10 % of retail pork samples test positive for HEV. However, the potential role of pig manure as a reservoir contributing to the environmental and zoonotic transmission of HEV genotype 3 remains insufficiently characterized.

To assess HEV contamination in manure, 61 pig slurry samples were collected from various collection points in northwestern Germany and analyzed for HEV and hepatitis A virus (HAV) RNA using quantitative PCR. In line with the “One Health” concept, which emphasizes the interconnectedness of humans, animals, and the environment, this study aimed to evaluate pig manure as a potential source of HEV contamination of agricultural land and food plants, thus posing a risk to both omnivorous and vegetarian populations.

HEV RNA was detected in 67 % (41/61) of the samples, with viral loads ranging from 5.2 × 10^2^ to 1.8 × 10^5^ copies/mL (median 6.8 × 10^3^ copies/mL), whereas HAV RNA was not detected in any sample. Genotyping via nested PCR identified HEV genotype 3c, a subtype frequently detected in human infections in Germany. To further characterize the viral particles, four samples with the highest viral loads were subjected to linear density gradient ultracentrifugation. Two distinct fractions were identified, corresponding to enveloped particles (suggesting urinary origin) and non-enveloped particles (suggesting fecal origin).

In conclusion, this study provides evidence for the frequent presence of both enveloped and non-enveloped HEV particles in pig slurry in Germany. These findings highlight manure as a potential environmental reservoir for HEV and underscore the need for further studies to determine HEV infectivity in slurry and to assess its epidemiological significance within the One Health framework.

## 1 Introduction

Hepatitis E virus (HEV) is a non-enveloped, single-stranded RNA virus belonging to the family Hepeviridae, with eight known genotypes that have different host ranges and transmission routes [1]. The tropical genotypes 1 and 2 are limited to humans and are mainly spread through the fecal–oral route via contaminated drinking water, causing large outbreaks in areas with poor sanitation. In contrast, genotypes 3 and 4 are zoonotic, infecting a wide range of mammalian hosts and being the main cause of locally acquired HEV infections in high-income countries [1]. Transmission typically happens through eating undercooked or raw animal products or direct contact with infected animals. Genotypes 5–8, found in wild boar and camels, are considered to have limited relevance to human health [1].

Pigs are the main reservoir for HEV genotype 3 (gt3) infections in Europe, although other animals like wild boar, deer, and rabbits can also carry the virus [1]. In rabbits, a unique subtype—HEV genotype 3r—has developed through host adaptation. HEV RNA has also been found in goats, sheep, and shellfish [[2], [3], [4], [5]]. Companion animals such as dogs, cats, and horses rarely have active HEV infections; however, serological evidence from Germany shows that 10 %, 6 %, and 2 % of these species [6], respectively, have HEV-specific antibodies—indicating occasional, non-sustained “spillover” infections without significant epidemiological impact for transmission. In developed regions like Europe, North America, Australia, and Japan, genotype 3 is most common, whereas genotype 4 is seen more often in East Asia [1].

In addition to pork consumption, alternative transmission routes for HEV genotypes 3 and 4 have been reported, including transfusion of contaminated blood products [7,8], occupational animal contact [9], and ingestion of contaminated shellfish [10]. A German retail survey found HEV RNA in approximately 10 % of 131 pork-derived products [11], while 50–60 % of pigs in Germany were seropositive for HEV antibodies, and around 17 % of slaughtered pig livers tested positive for HEV RNA [12,13].

Environmental transmission routes are becoming more widely acknowledged. HEV RNA has been found in 0.6 % of ready-to-eat salads in Italy [14], strawberries in Canada [15], and 1.3 % of mixed berries in Ireland [16], raising concerns about contamination through agricultural water or manure. Additionally, high HEV detection rates have been reported in pig slurry across several countries—75 % in Italian farms (genotype 3f) [17], as well as in Brazil [18] and the United States [19]—indicating that livestock waste could be an ongoing source of viral spread in the environment.

Given the frequent detection of HEV in pig manure and its use as organic fertilizer, understanding its role in environmental contamination and human exposure is crucial within the One Health framework. The close connection between livestock production, environmental management, and food safety emphasizes the need for integrated surveillance of zoonotic viruses. Therefore, this study aimed to determine the prevalence of HEV RNA in pig slurry samples collected in Lower Saxony, northern Germany, from 2022 to 2024 using quantitative PCR (qPCR), including detection of Hepatitis A virus (HAV) for comparison. Samples with the highest HEV RNA levels were further analyzed by density gradient ultracentrifugation to characterize viral particle properties.

From a One Health perspective, characterizing HEV occurrence and particle stability in pig manure provides essential insight into the intersection of animal, environmental, and human health. These findings may inform risk assessment strategies, guide safe agricultural practices, and support the development of integrated monitoring systems to minimize zoonotic HEV transmission through the food chain.

## 2 Materials and methods

### 2.1 Origin and handling of slurry samples

34 pig slurry samples, 50 % from single farmers and 50 % from pooled storage containers, obtained in October 2022, and 27 samples, 100 % from single farmers, obtained in July 2024, originated from various collection points in the north-western region of Germany. They were transported under refrigerated (4 °C) conditions to an accredited service laboratory of the Lower Saxony Chamber of Agriculture (*LUFA = “Landwirtschaftliche Untersuchungs- und Forschungsanstalt”*). Each sample of the slurry was labeled with ongoing numbers and bottled either in a 250 mL polyethylene container with a sealing cap or 50 mL Falcon tubes and stored under 4 °C. 1 mL of each of the 61 samples were centrifuged and stored at −20 °C for further analysis.

### 2.2 RNA extraction, Real-Time Polymerase Chain Reaction (qRT-PCR), Nested PCR

Viral RNA was extracted from a sample volume of 200 μL using the QiAmp MiniElute Virus Spin Kit (Qiagen, Hilden, Germany). HEV RNA copies were quantified by one-step quantitative real-time PCR (qRT-PCR) (ViiA™ 7 Real-Time PCR System Life Technologies, Carlsbad, CA) with HEV specific primers and probes (JVHEVF 5′GGTGGTTTCTGGGGTGAC; JVHEVR 5′AGGGGTTGGTTGGATGAA, TaqMan minor grove binding probe JVHEVPh 5′FAM-TGATTCTCAGCCCTTCGC-MGB). Cycling conditions were as follows: initiation step at 50 °C for 5 min, 40 cycles of amplification with each cycle consisting of 95 °C for 3 s and 60 °C for 30 s. Serial dilutions of an HEV-containing plasmid were used as a standard for quantification. The lower limit of detection (LLoD) was set to cycle 40 (5 × 10^2^ copies/ml). HAV levels were quantified using the AltoStar® HAV RT-PCR Kit (Altona Diagnostics, Hamburg, Germany) according to manufacturer protocol. Isolated viral RNA from selected slurry samples were sequenced using a nested genotyping protocol and primer set MJ-C as described previously [20].

### 2.3 Linear iodixanol gradient centrifugation

To determine the density and therefore the biological feature of the HEV particles in the manure samples, a linear iodixanol gradient centrifugation technique was used as described previously with several modifications [21]. Briefly, 250 μL of a pig slurry sample was first centrifuged through a 0.22 μm ultrafiltration unit to eliminate insoluble debris. The flow through was pipetted on top of an 8–40 % linear iodixanol/sucrose (OptiPrep, Progen Biotechnik, Heidelberg, Germany) gradient which was ultracentrifuged in a SW55 Ti rotor (Beckmann Coulter, Krefeld, Germany) at 43200 rpm and 4 °C for 18 h. 20 fractions á 250 μL were taken from bottom to top for each sample analyzed and the density was determined by refractometry. Viral RNA was purified from each fraction using the QiAmp MiniElute Virus Spin Kit (Qiagen) followed by qRT-PCR to quantify HEV RNA copy numbers as described above.

## 3 Results

**Fig. 1:**
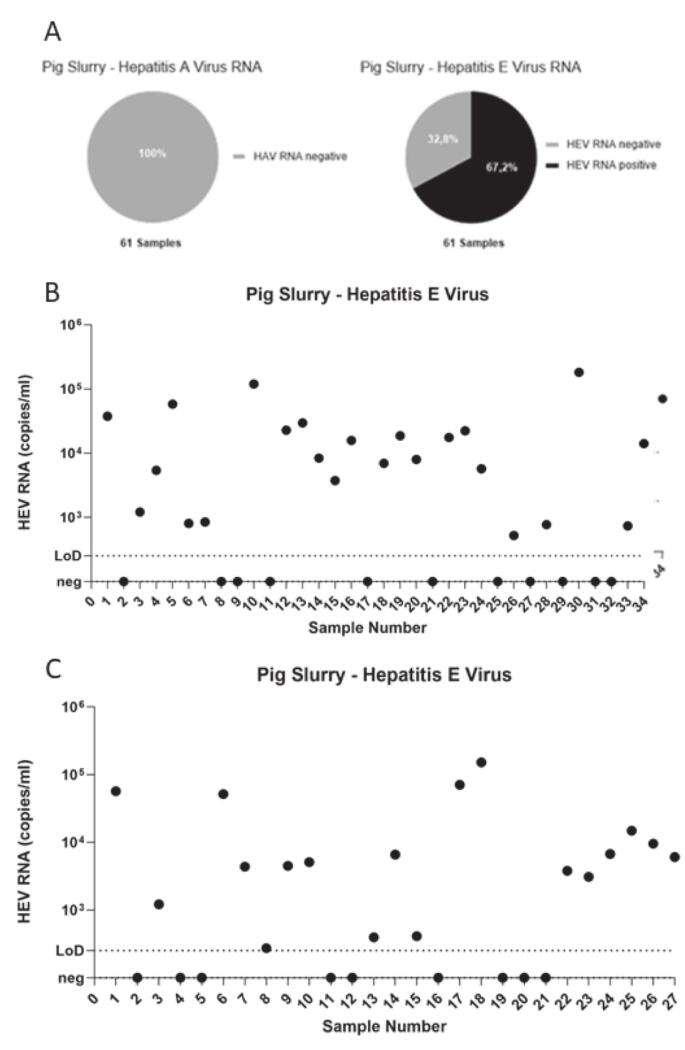
**Contamination of pig slurry samples with Hepatitis E virus.** (A) Overview of the tested samples by RT-PCR for Hepatitis A and E virus and the percentage of positive and negative samples. (B) 34 pig slurry samples (August 2022) were tested for HEV RNA copies/mL by a one-step qRT-PCR (black dots, the upper dotted line represents *Lower Limit of Quantification* (= LLoQ) = 500 copies/mL, lower dotted line represents *undetermined*). Samples with the highest titer are marked with a light and a dark triangle. (C) 27 pig slurry samples (July 2024) were tested for HEV RNA copies/mL by a one-step qRT-PCR (black dots, the dotted line represents *Lower Limit of Detection* (= LLoQ) = 500 copies/mL).

While 41 of 61 (67 %) samples tested positive for HEV RNA, with viral titers ranging from 5.2 × 10^2^ to 1.8 × 10^5^ HEV RNA copies/mL in pig manure suspension (Fig. 1A/B/C), none of the 61 samples tested positive for HAV RNA (0 %). The rate of HEV positivity in samples from individual farmers (30 of 44; 68 %) was not significantly different from the rate in pooled samples from multiple farms (11 of 17; 65 %). The HEV viral load in samples from individual farms and pooled farm samples did not differ significantly (*p* = 0.313, Mann-Whitney test).

**Fig. 2:**
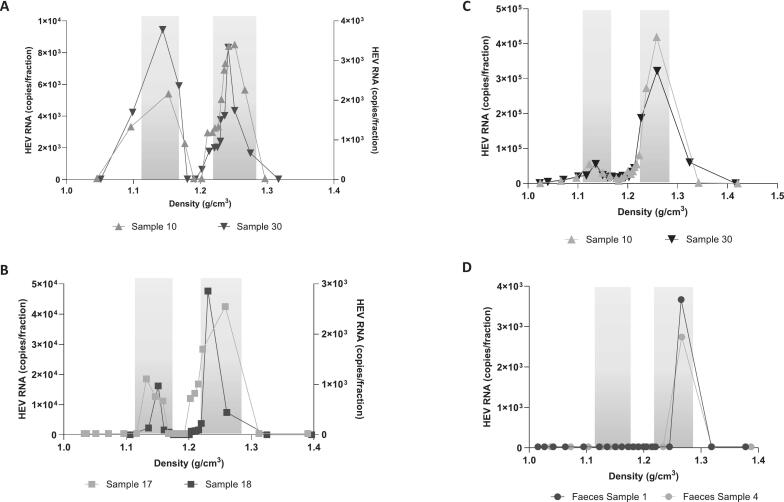
**Characteristics of HEV particles in pig slurry.** A. Distribution of the HEV titers over density from two samples after subjection over a linear iodixanol/sucrose gradient. B. Distribution of the HEV titers over density from two other samples after subjection over a linear iodixanol/sucrose gradient. C. and D. Samples from A. and B. were subjected to a gradient centrifugation 1.5 years after storage under 4 °C. The left grey box indicates the density distribution for the quasi-enveloped form of HEV, whereas the right grey box indicates the naked form of HEV.

To gain a more in-depth understanding of the biological characteristics of the manure, two samples from each time point with the highest viral load (see Fig. 1B or 1C) were analyzed using ultracentrifugation with a linear iodixanol/sucrose gradient. All four samples showed the presence of both the quasi-enveloped (ρ = 1.11–1.15 g/cm^3^) and the naked (ρ = 1.22–1.26 g/cm^3^) forms of Hepatitis E viral particles (Fig. 2A/B; indicated by light grey boxes), confirming a mixture of urine (quasi-enveloped) and feces (naked) in the pig manure. The peak titer points of the quasi-enveloped and naked samples were then subjected to nested PCR to determine their genotype. Using an online tool (Genome Detective) and phylogenetic analysis, all four cases were identified as Hepatitis genotype 3c. Additionally, the same samples from Fig. 2A, which were stored at 4 °C for 18 months, were reanalyzed to assess the density of HEV-positive manure samples after long-term storage. Only a minor peak for the enveloped virus was detected, while most HEV particles were naked, with a density around 1.22–1.26 g/cm^3^ (Fig. 2C). As controls, two of the four positive samples from pure pig feces were subjected to gradient analysis. Only a single fraction in each sample matched the density of naked HEV particles, similar to those found in HEV-positive human stool samples (Fig. 2D). Overall, the characteristics of manure and pig feces are comparable to human HEV-positive serum and stool samples.

## 4 Discussion

The foodborne transmission of Hepatitis E virus (HEV) genotype 3 through the consumption of contaminated pork products is well established across Europe [[22], [23], [24], [25], [26]]. In contrast, in low- and middle-income countries where genotypes 1 and 2 dominate, HEV infections are mainly waterborne and linked to outbreaks involving contaminated drinking water [1]. The present study offers the first evidence that HEV can be detected in roughly two-thirds of pig manure samples collected in northern Germany, expanding the current understanding of HEV ecology and transmission routes in industrialized regions.

Until now, HEV research in Europe has mainly focused on zoonotic transmission through the consumption of pork, game meat, rabbit, or shellfish [1,22,[27], [28], [29]]. The findings of this study suggest that pig feces—and, consequently, manure used as agricultural fertilizer—may represent an additional environmental route of HEV transmission. This environmental pathway could plausibly explain the repeated detection of HEV RNA in fruits and vegetables reported in several European countries [14,16]. Although the infectivity of HEV RNA found in manure has not yet been demonstrated, its persistence indicates a potential risk, especially for individuals who do not eat meat. Crops fertilized with untreated manure or eaten raw could serve as an indirect reservoir for human exposure.

The detection of HEV RNA in about 67 % of slurry samples at two separate times (2022 and 2024), whether from individual farms or pooled collections, indicates a steady level of contamination in northwestern Germany. Although the exact geographic origin of positive samples couldn't be disclosed to protect farm confidentiality, the consistent detection over the years shows that HEV presence in pig manure is a persistent, regional issue rather than a random occurrence.

These results highlight the need to reevaluate current models of HEV transmission. In addition to traditional zoonotic transmission through animal products, finding HEV RNA in manure stresses an environmental link between livestock farming, agriculture, and human exposure. This stresses the importance of the One Health approach, which combines animal health, environmental care, and human public health to tackle complex zoonotic risks.

Further insight was gained through density gradient ultracentrifugation of high-titer samples. The identification of both enveloped (urine-derived) and non-enveloped (feces-derived) HEV particles shows the coexistence of structurally different viral forms within pig slurry. The presence of intact viral particles indicates that at least some HEV RNA detected probably comes from complete virions with potential infectivity.

Despite its novelty, the present study has several limitations. (i) The infectivity of HEV particles in pig slurry still needs to be confirmed through cell culture or animal infection models. (ii) The regional distribution and seasonality of HEV in manure across Germany and Europe require systematic surveillance. (iii) The extraction protocol used was not optimized for environmental matrices, which suggests that more sensitive methods could reveal even higher viral loads or lead to a higher rate of positivity.

## 5 Conclusion

This study shows that HEV RNA is detectable in two-thirds of pig manure samples from northern Germany. Although the infectivity of these particles is still unknown, the data strongly suggest that pig manure is a significant, previously overlooked component in the environmental persistence and transmission of HEV. These findings help explain the recurrent detection of HEV in fruits and vegetables in non-endemic regions with high sanitation standards, identifying pig manure as a plausible intermediary reservoir—the “missing link” connecting animal infection, environmental contamination, and human exposure.

### 5.1 Public health and one health implications

From a One Health perspective, detecting HEV RNA in pig manure has significant implications for surveillance and risk reduction. Regularly monitoring manure used in farming, combined with implementing standardized treatment or composting protocols to deactivate viral pathogens, could greatly lower environmental contamination. Improved biosecurity on farms and increased awareness among agricultural workers and veterinarians should support these efforts. Adding environmental HEV testing to current zoonotic surveillance programs would offer valuable information for risk assessment and help shape evidence-based policies. This interdisciplinary cooperation among veterinary, ecological, and human health sectors is crucial to reduce the risk of HEV spread through the food chain.

## CRediT authorship contribution statement

**Florian Hinte:** Writing – original draft, Validation, Software, Methodology, Investigation, Formal analysis, Data curation, Conceptualization. **Marc Lütgehetmann:** Writing – review & editing, Visualization, Supervision, Methodology. **Toni Luise Meister:** Writing – review & editing, Methodology, Formal analysis. **Katja Giersch:** Visualization, Software, Investigation, Formal analysis. **Maura Dandri:** Writing – review & editing, Supervision, Software, Resources. **Julian Schulze zur Wiesch:** Writing – review & editing, Writing – original draft, Supervision, Data curation. **Sven Pischke:** Writing – review & editing, Writing – original draft, Validation, Supervision, Funding acquisition, Formal analysis, Data curation, Conceptualization.

## Funding

F.H., M.L., M.D., J.SzW. and S.P. are supported by 10.13039/100009139German Center for Infection Research (DZIF, TTU TTU 05.328, T.L.M., M.L., M.D., J.SzW are supported by the 10.13039/100009139German Center for Infection Research (DZIF, TTU 01.719 and 05.823). associated with the DFG collaborative research center 1648 (SFB 1648/1 2024–512741711).

## Declaration of competing interest

None of the authors has a conflict of interest concerning this manuscript.

## Data Availability

Data will be made available on request.
